# Hemodynamic response during endotracheal suctioning predicts awakening and functional outcome in subarachnoid hemorrhage patients

**DOI:** 10.1186/s13054-020-03089-w

**Published:** 2020-07-14

**Authors:** Verena Rass, Bogdan-Andrei Ianosi, Anna Lindner, Mario Kofler, Alois J. Schiefecker, Bettina Pfausler, Ronny Beer, Erich Schmutzhard, Raimund Helbok

**Affiliations:** 1 Department of Neurology, Medical University of Innsbruck, Anichstrasse 35, 6020 Innsbruck, Austria; 2 Institute of Medical Informatics, UMIT, University for Health Sciences, Medical Informatics and Technology, Eduard Wallnoefer-Zentrum 1, 6060 Hall, Austria

**Keywords:** Subarachnoid hemorrhage, Brainstem integrity, Autonomic testing, Critical care, Endotracheal suctioning

## Abstract

**Background:**

Endotracheal suctioning (ES) provokes a cumulative hemodynamic response by activation of sympathetic and parasympathetic circuits in the central nervous system. In this proof-of-concept study, we aimed to analyze hemodynamic changes during ES in ventilated subarachnoid hemorrhage (SAH) patients and investigated whether the associated hemodynamic changes relate to the time to arousal and functional outcome.

**Methods:**

For the current observational study, 191 SAH patients admitted to the neurological intensive care unit of a tertiary hospital requiring mechanical ventilation were included. One thousand eighty ES episodes during the first 72 h of admission were analyzed. Baseline median heart rate (HR) and mean arterial pressure (MAP) were compared to peak HR and MAP during ES based on 5-min averaged data (ΔHR and ΔMAP). Multivariable analysis to assess associations between ΔHR and ΔMAP and time to arousal (time to Richmond Agitation Sedation Scale ≥ 0, RASS) and poor functional outcome (modified Rankin Scale Score > 2, mRS) was performed using generalized estimating equations.

**Results:**

Patients were 59 (IQR, 50–70) years old and presented with a median admission H&H grade of 4 (IQR, 3–5). In-hospital mortality was 22% (25% at 3 months) and median time to arousal was 13 (IQR, 4–21) days. HR increased by 2.3 ± 7.1 beats per minute (bpm) from 75.1 ± 14.8 bpm at baseline. MAP increased by 3.2 ± 7.8 mmHg from baseline 80.9 ± 9.8 mmHg. In multivariable analysis, ΔHR (*p* < 0.001) was significantly lower in patients who regained consciousness at a later time point and a lower ΔHR was associated with poor functional 3-month outcome independent of RASS (adjOR = 0.95; 95% CI = 0.93–0.98) or midazolam dose (adjOR = 0.96; 95% CI = 0.94–0.98). ΔMAP was neither associated with the time to regain consciousness (*p* = 0.087) nor with functional outcome (*p* = 0.263).

**Conclusion:**

Augmentation in heart rate may quantify the hemodynamic response during endotracheal suctioning in brain-injured patients. The value as a biomarker to early discriminate the time to arousal and functional outcome in acutely brain-injured patients needs prospective confirmation.

## Background

Despite tremendous improvements in the neurocritical care management with reduced case fatalities over the past decades, subarachnoid hemorrhage (SAH) is still a devastating disease with a high rate of disability and mortality [1]. Prediction of outcome and in specific estimating the time to arousal remains challenging. Especially in the early phase after SAH, most patients with severe disease need sedative drugs, which renders a proper clinical examination difficult [2]. Separating pharmacological induced coma from altered consciousness due to structural damage would help to discriminate those patients who are likely to regain consciousness early from those with prolonged coma. In a recent prospective study using surface electroencephalography (EEG) monitoring, it was possible to identify 15% of unresponsive patients with a dissociation between brain activity and commanded motor behavior [3]. The authors provided spoken commands and found that patients with EEG activity despite missing motor behavior to commands had better outcomes than those without brain activity.

Endotracheal suctioning (ES) is routinely performed in mechanically ventilated patients to prevent airway obstruction. The nociceptive stimulus usually triggers the cough reflex [4] and leads to sympathetic activity resulting in blood pressure and heart rate increase [5–8]. In contrast, vagal parasympathetic activation may provoke bradycardia and hypotension [9]. The hemodynamic response during ES reflects the net effect of those mechanisms. The extent of hemodynamic changes may give insight in functionality of neuronal circuits and may also help to identify patients with less neuronal injury who are likely to wake up.

In this study, we sought to study the physiologic response to ES by quantifying the hemodynamic response measuring changes in heart rate (HR) and mean arterial blood pressure (MAP). The main hypothesis was that patients who exhibit a less pronounced hemodynamic response during ES are more severely injured and have a prolonged comatose state and worse outcome when compared to patients with a more pronounced response.

## Methods

### Study design, setting, and patient selection

**Fig. 1:**
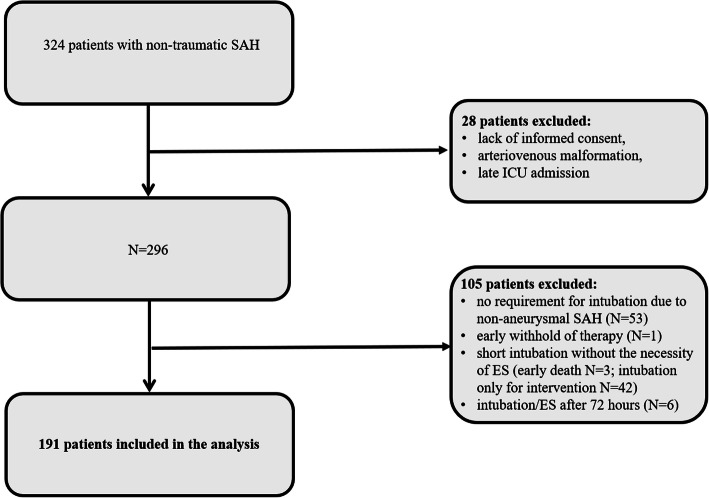
Flow chart showing the patient selection

The study design was guided by the STROBE statement on observational studies. This is a retrospective analysis of prospectively collected data of patients with non-traumatic SAH that were admitted to the neurological intensive care unit of a tertiary care hospital (Medical University of Innsbruck) between 2010 and 2017. Inclusion criteria encompassed (1) non-traumatic SAH, (2) age ≥ 18 years, and (3) intensive care unit stay for at least 24 h. Out of 324 screened patients, 28 patients were excluded due to (1) lack of informed consent, (2) arteriovenous malformation, and (3) late admission (> 7 days after bleeding onset) resulting in 296 patients included in our registry. Out of these, 105 patients were excluded due to (1) no requirement for aneurysm intervention and intubation due to non-aneurysmal SAH (*N* = 53) or early withhold of therapy (*N* = 1), (2) short intubation period without the necessity of ES (early death *N* = 3; intubation only for intervention *N* = 42), or (3) intubation/ES after 72 h (*N* = 6) leaving 191 eligible patients for the current analysis (Fig. 1). All patients without the detection of an aneurysm in repeated angiography fulfilled the clinical and radiographic criteria for spontaneous SAH suggestive of aneurysmal SAH with more blood compared to a typical perimesencephalic SAH. The conduct of the study was approved by the local ethics committee (Medical University of Innsbruck, AM4091-292/4.6). Written informed consent was obtained according to local regulations.

### Patient management and grading

Critical care management adhered to international guidelines [10, 11] with the exception of nimodipine which was prophylactically administered intravenously and not orally in poor-grade patients. Clinical grading was done by using the Hunt and Hess (H&H) score at initial presentation after the bleeding without sedation [12]. Admission computed tomography (CT) scans were scored by a neuroradiologist blinded to clinical data using the modified Fisher (mFisher) score and SEBES (Subarachnoid Hemorrhage Early Brain Edema Score) [13, 14]. Magnetic resonance imaging (MRI) was done when clinically indicated. Ruptured aneurysms were secured by either clipping or coiling after discussion in an interdisciplinary team. Transcranial color-coded duplex sonography (TCD, LOGIQ S8; GE Healthcare, Chicago, IL) was regularly performed in order to follow patients for vasospasm. Vasospasm was defined as elevation of mean velocities greater than 120 cm/s in the middle or anterior cerebral artery or daily change in mean TCD velocities greater than 50 cm/s. Severe vasospasm (> 200 cm/s) was further confirmed by catheter cerebral angiogram. The definition of delayed cerebral ischemia (DCI) was based either on clinical deterioration with a new focal neurologic deficit, a decrease of greater than or equal to 2 points on the Glasgow Coma Scale, or a new infarct on the CT or MRI scan not attributable to other causes [15]. Severe vasospasm or DCI was treated with induced hypertension. When clinical symptoms were refractory to hypertensive treatment or neuromonitoring variables were indicative of further deterioration, cerebral pan-angiography was pursued to evaluate for intra-arterial nimodipine treatment. Hemodynamic augmentation was achieved using vasopressors (most commonly noradrenaline or in the setting of stunned myocardium or low cardiac output dobutamine was used) or volume administration. Sedation and analgesia consisted of continuous IV infusions of midazolam and/or sufentanil at the discretion of the treating physician (Table 1). Generally, a pressure-regulated volume-controlled ventilation mode was used. To wean patients off, assisted spontaneous breathing with pressure support and continuous positive airway pressure (CPAP) was used. Targeted temperature management with the aim of temperatures between 36.5 and 37.5 °C and fever prevention was applied. Endotracheal suctioning was routinely performed during the stay in the ICU to ensure secretion clearing and to maintain airway patency. Preoxygenation before ES was done in all patients.

**Table 1:** Mean daily dosage of sedatives, analgesics, catecholamines, and atropine during the study period (first 72 h after admission)

Medication	Dosage; mean daily (±SD)	*N* (%)
Sufentanil [μg]	4073 (± 2487)	164 (86%)
Remifentanil [mg]	4 (± 3)	14 (7%)
Midazolam [mg]	407 (± 264)	172 (90%)
Propofol [mg]	497 (± 601)	57 (30%)
Noradrenaline [mg]	13 (± 9)	179 (94%)
Dobutamine [mg]	203 (± 112)	120 (63%)
Atropine [mg]	1.3 (± 1.0)	31 (16%)

### Data collection

Patient demographics, hospital complications, and outcomes were prospectively collected and discussed in weekly meetings held by the study team and treating neurointensivists.

Three-minute median values of vital parameters including HR and MAP were automatically saved in the electronic patient data management system (PDMS, CentricityTM Critical Care 8.1 SP7; GE Healthcare Information Technology, Dornstadt, Germany). Due to the inconsistent data entry in the storage system with seconds apart, we chose to calculate 5-min interval data based on the mean as a post hoc analysis. Arterial blood pressure was most often measured through either radial or femoral arterial lines; HR was measured through an ECG or an arterial line. Continuous ECG recordings were performed in all patients.

Only the first 72 h of admission were analyzed in order to avoid a selection bias of poor-grade patients requiring longer intubation and to allow early discrimination of patients. The day of admission was denoted as day 1 (first 24 h of admission). The sedation status was retrospectively assessed by chart review including daily notes of nurses, daily notes of treating neurointensivists, and the medical report using the RASS (Richmond Agitation Sedation Scale) [16, 17]. The 10-point scale was classified as follows: − 5 or − 4 (deep sedation), − 3 to − 1 (moderate to mild sedation), and 0–5 (no sedation). The record of RASS was missing in 12 patients. Moreover, the daily cumulative dose of midazolam in milligram was calculated.

### Outcome measures

The primary outcome measure was time to arousal defined as the time to RASS ≥ 0. Secondary deterioration was not counted if the patient was conscious (RASS ≥ 0) for at least 5 days. Data were missing in 6 patients due to early repatriation (*N* = 4) or missing documentation (*N* = 2). Functional outcome after 3 months was assessed by a study nurse blinded to the clinical course of the patient via telephone interview using the modified Rankin Scale Score (mRS). Poor outcome was defined as a mRS > 2. In 10 patients who were lost to follow-up, the discharge mRS was carried forward to 3 months.

### Data management and statistical analysis

Unreliable systolic (< 40 mmHg or > 250 mmHg; 13 measurements) or diastolic (< 15 mmHg) blood pressure values secondary to transducer flushing, movement artifacts, connection-reconnection artifacts or obstructed arterial lines were deleted. Timing of suctioning episodes was identified in the PDMS. Baseline HR or MAP was calculated by the median HR or MAP between 30 and 10 min before ES. Thirty minutes before and after ES were analyzed. If the gap between two episodes of ES was less than 1 h, the latter was discarded. ∆HR and ∆MAP at each time point were calculated by subtracting HR or MAP at baseline from the HR or MAP during suctioning or the following 30 min. Ten percent increase of HF and MAP at ES was judged significant.

Continuous variables were expressed as mean ± SD or median and interquartile range (IQR). Categorical variables were reported as counts and proportions. Univariate and multivariable analysis was performed with generalized estimating equation models (GEE) with the correlation matrix best fitting the data as proposed by Chan et al. based on the lowest Quasi Likelihood under Independence Model Criterion (QIC) and Corrected Quasi Likelihood under Independence Model Criterion (QICC) in order to account for repeated measurements within one patient [18]. All multivariable models were adjusted for predefined variables including H&H grade (to account for disease severity), age (due to less pronounced autonomic responses in older subjects [19]), RASS or daily cumulative midazolam dose (to account for sedation depth), daily cumulative dose of sufentanil, dobutamine, and noradrenaline and the absolute HR or MAP, and indicated appropriately. Due to the strong influence of vigilance by benzodiazepines and resulting probable collinearity between RASS and midazolam, we built two distinct models including each variable separately. Cases with missing values were included. Adjusted odds ratios (adjORs) with 95% confidence intervals (CI) were calculated. The level of significance was set at *α* = 0.05. All analyses and graphical representations were performed with IBM-SPSS V24.0 (SPSS Inc., Armonk, NY) and Prism 5 for Windows V5.01 (GraphPad Software, Inc., LA Jolla, CA).

## Results

Of 324 screened SAH patients, 191 met the inclusion criteria. Detailed information on demographics, hospital complications, and outcomes is given in Table 2.

**Table 2:** Baseline characteristics, complications and outcomes

Clinical characteristics	*N* = 191
Age [years]	59 (50–70)
Female sex	129 (68%)
H&H grade after bleeding	
1–3	86 (45%)
4–5	105 (55%)
Pre-existing hypertension	81 (42%)
Diabetes mellitus	15 (8%)
LOC at ictus	103 (54%)
Admission radiological characteristics	
Modified Fisher scale	
1	8 (4%)
2	18 (9%)
3	39 (20%)
4	126 (66%)
SEBES (low-grade, 0–2)	114 (61%)
SEBES (high-grade, 3–4)	74 (40%)
ICH present on admission CT scan	56 (29%)
Hydrocephalus requiring EVD placement	134 (70%)
Aneurysm size [mm]	6 (4–8)
Aneurysm treatment	
Endovascular coiling	106 (56%)
Neurosurgical clipping	59 (31%)
Withhold therapy	16 (8%)
Non-aneurysmal SAH^*^	9 (5%)
Complications	
Pneumonia	110 (58%)
Urinary tract infection	50 (26%)
Ventriculitis	30 (16%)
Vasospasm	103 (54%)
DCI	42 (22%)
Intubated days	10 (3–17)
H&H grades 1–3	5 (2–15)
H&H grades 4–5	12 (5–20)
Outcome Characteristics	
Length of ICU stay [days]	23 (13–34)
In-hospital mortality	42 (22%)
3-month mRS	
0	12 (6%)
1	35 (18%)
2	26 (14%)
3	17 (9%)
4	17 (9%)
5	36 (19%)
6	48 (25%)

*SAH* subarachnoid hemorrhage, *ES* endotracheal suctioning, *H&H* Hunt and Hess, *LOC* loss of consciousness, *ICH* intracerebral hemorrhage, *EVD* external ventricular drain, *CT* computed tomography, *SEBES* Subarachnoid Hemorrhage Early Brain Edema Score, *DCI* delayed cerebral ischemia, *mRS* modified Rankin Scale. Data are given in median (IQR) and counts (%)

*Patients were aneurysm negative in repeated cerebral angiogram 2–3 weeks apart

Among patients in whom suctioning was performed, all clinical severity grades were included with 18 (9%) patients having an admission H&H-grade of 1, 27 (14%) H&H 2, 41 (22%) H&H 3, 16 (8%) H&H 4, and 89 (47%) H&H 5. Patients were mechanically ventilated for a median of 10 (IQR, 3–17) days ranging from 1 to 40 days. Overall, 1080 ES episodes within 72 h of hospitalization were analyzed with a median of 5 (IQR, 3–8) suctioning episodes per patient. In these patients, median 2 (IQR, 1–3) suctioning episodes per day were performed.

Majority of ventilation modes during ES were a controlled ventilation mode (71%) followed by assisted ventilation (29%).

### Hemodynamic response during suctioning

**Fig. 2:**
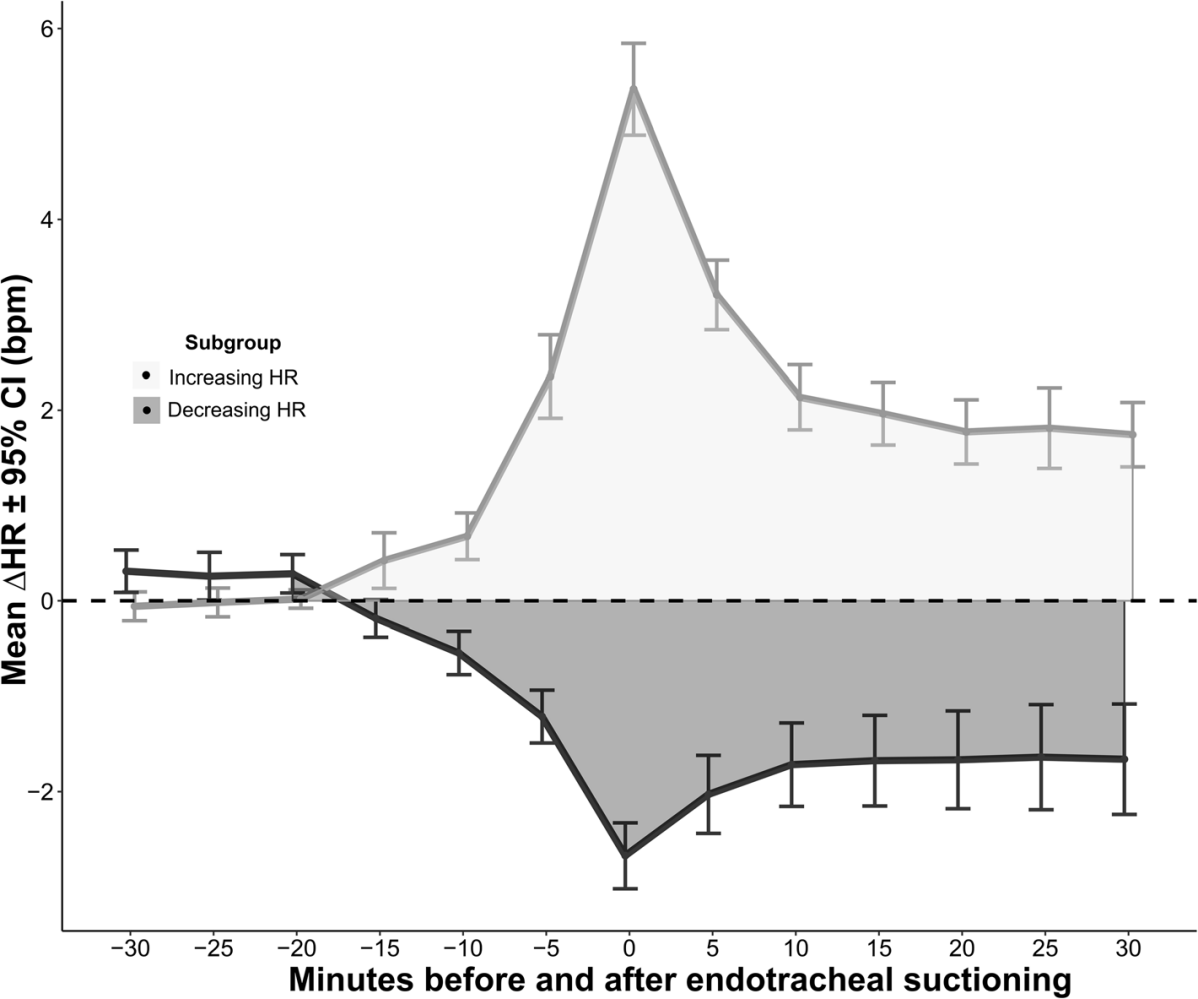
Increases in heart rate (HR) occurred in 63% and decreases in 37% of episodes of endotracheal suctioning (N=1080)

As a response to ES, overall HR increased by mean 2.3 ± 7.1 bpm (p < 0.001) from 75.1 ± 14.8 bpm at baseline which corresponded to an increase of 3.5 ± 10.0%. During 177/1080 (16%) suctioning episodes, a significant increase of > 10% was reached. HR increased in 681/1080 (63%; +5.3 ± 6.8) and decreased in 399/1080 37% (− 2.8 ± 4.4) episodes (Fig. 2). MAP increased by mean 3.2 ± 7.8 mmHg (*p* < 0.001; 4.1 ± 9.9%) from 80.9 ± 9.8 mmHg at baseline.

Poor-grade patients (H&H grades 4 & 5) showed significantly lower increases in HR during suctioning as compared to good-grade patients (H&H grades 1–3, *p* < 0.001). However, no difference in MAP (*p* = 0.435) was observed. Neuroradiographic parameters on admission did not well discriminate between suctioning associated ∆HR (intracerebral hemorrhage on admission, *p* = 0.192; low-/high-grade SEBES, *p* = 0.092; low-/high-grade mFisher, *p* = 0.196). Lower ∆MAP was only associated with high-grade SEBES (*p* = 0.025) but not with low- or high-grade mFisher (*p* = 0.101) or ICH present on admission CT scan (*p* = 0.162; Table 3).

**Table 3:** Hemodynamic response during endotracheal suctioning

Variable	HR change [bpm]	***p*** value	MAP change [mmHg]	***p*** value
H&H grades 1–3	3.3 (± 6.9)	< 0.001	3.0 (± 7.8)	0.435
H&H grades 4–5	1.5 (± 7.2)		3.3 (± 7.9)	
No ICH on admission	2.1 (± 6.8)	0.192	2.9 (± 7.6)	0.162
ICH on admission	2.7 (± 8.0)		3.7 (± 8.2)	
Low-grade SEBES (0–2)	2.5 (± 7.2)	0.092	3.5 (± 7.8)	0.025
High-grade SEBES (3–4)	1.8 (± 7.2)		2.4 (± 7.8)	
Low-grade mFisher (1–2)	2.9 (± 6.4)	0.196	2.4 (± 6.3)	0.101
High-grade mFisher (3–4)	2.2 (± 7.3)		3.3 (± 8.1)	
Controlled ventilation mode	1.1 (± 6.0)	< 0.001	2.6 (± 7.3)	0.004
Assisted ventilation mode	5.3 (± 8.8)		4.4 (± 8.9)	

Data are given in mean (± standard deviation)

All models were calculated with univariate GEE models

*Bpm* beats per minute, *H&H* Hunt and Hess, *ICH* intracerebral hemorrhage, *SEBES* Subarachnoid Hemorrhage Early Brain Edema Score, *mFisher* modified Fisher score

The response of HR (*p* < 0.001) and MAP (*p* = 0.004) was significantly lower during controlled ventilation mode as compared to assisted ventilation (Table 3). Importantly, during most ES episodes (829/1080, 83%), patients were deeply sedated (RASS − 5 or − 4). In 46 (4%) episodes, a RASS between − 3 and − 1; in 54 (5%), a RASS between 0 and 1; and in only 11 (1%), a RASS between 2 and 4 were recorded. RASS was missing for 77 (7%) episodes.

### Hemodynamic response during suctioning and time to arousal

Time to arousal was median 13 (IQR, 4–21) days (*N* = 142). Five patients remained in a vegetative state (mean ∆HR, 0.3 ± 4.0). Out of 42 patients who died, 38 did not regain consciousness before death (mean ∆HR, 1.9 ± 7.7). ∆HR (B = − 0.191, Wald statistic = 25.4, df = 1, *p* < 0.001) during ES was significantly lower in patients with prolonged time to regain consciousness in a model adjusted for H&H grade, age, and absolute HR. Even when additionally correcting for daily cumulative dose of dobutamine, noradrenaline, sufentanil, and RASS (B = − 0.130, Wald statistic = 12.5, df = 1, *p* < 0.001) or cumulative midazolam dose (B = − 0.166, Wald statistic = 20.4, df = 1, *p* < 0.001), this association remained robust. ∆MAP (*p* = 0.087) was not associated with time to arousal.

### Hemodynamic response during suctioning and 3-month outcome

**Fig. 3:**
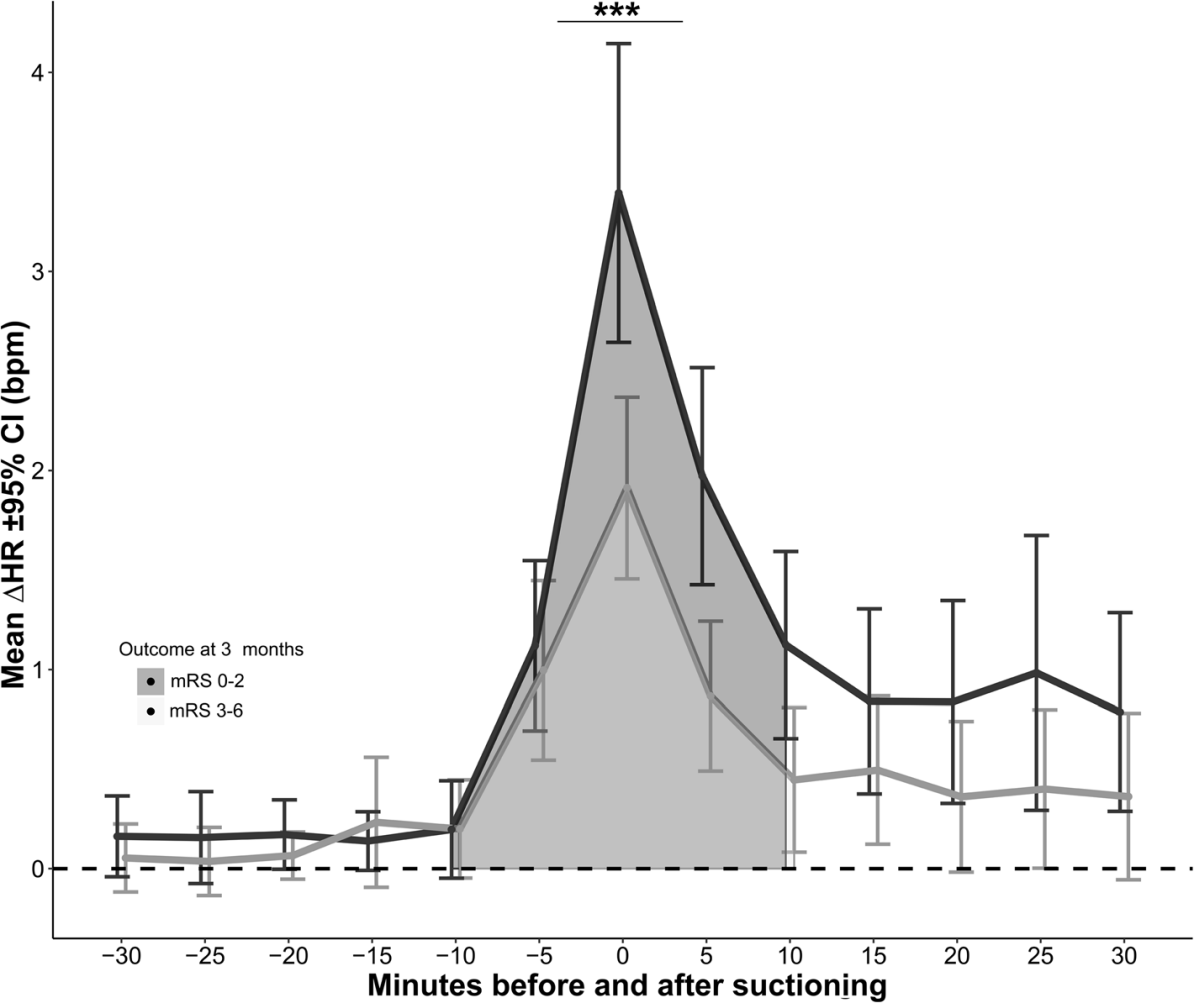
The increase of heart rate (HR) during endotracheal suctioning (total *N*=1080 epidsodes) was significantly lower in patients with poor functional outcome at 3 months as compared to patients with good outcome (*p* < 0.001). mRS, modified Rankin Scale Score

∆HR was significantly lower in patients with poor 3-month functional outcome as compared to those with good functional outcome (1.7 ± 6.9 vs. 3.3 ± 7.5; *p* = 0.001; Fig. 3). In multivariable analysis adjusted for admission H&H grade; age; absolute HR; daily cumulative dose of sufentanil, dobutamine, and noradrenaline; and daily cumulative midazolam dose or RASS, ∆HR was still significantly lower in patients with poor outcome (*p* < 0.001; Table 4). When separating in increasing and decreasing responses of HR, only a positive ∆HR (*p* < 0.001) but not negative ∆HR (*p* = 0.728) was associated with poor outcome.

**Table 4:** Association between increase of heart rate during endotracheal suctioning and poor functional 3-month outcome

	Adjusted for midazolam dose (model 1)		Adjusted for RASS* (model 2)	
Variables	AdjOR, 95% CI	***p*** value	AdjOR, 95%-CI	***p*** value
∆HR [bpm]	0.96, 0.94–0.98	< 0.001	0.95, 0.93–0.98	< 0.001
Age [years]	1.06, 1.05–1.07	< 0.001	1.06, 1.05–1.07	< 0.001
H&H grade	1.86, 1.65–2.09	< 0.001	1.95, 1.72–2.22	< 0.001
Absolute HR [bpm]	1.02, 1.01–1.03	0.003	1.02, 1.01–1.03	< 0.001
Daily cumulative dobutamine dose [mg]	0.998, 0.997–0.999	0.001	0.998, 0.997–0.999	< 0.001
Daily cumulative noradrenaline dose [mg]	0.99, 0.98–1.00	0.173	0.99, 0.97–0.998	0.028
Daily cumulative sufentanil dose [μg]	1.00, 1.00–1.00	0.074	1.00, 1.00–1.00	0.037
Daily cumulative midazolam dose [mg]	1.00, 0.99–1.00	0.344	–	–
RASS	–	–	0.70, 0.50–0.98	0.040

*One hundred seventy-nine patients were included due to missing RASS recordings

*∆HR* change of heart rate during suctioning, *H&H* Hunt and Hess, *RASS* Richmond Agitation Sedation Scale, *adjOR* adjusted odds ratio, *CI* confidence interval

**Fig. 4:**
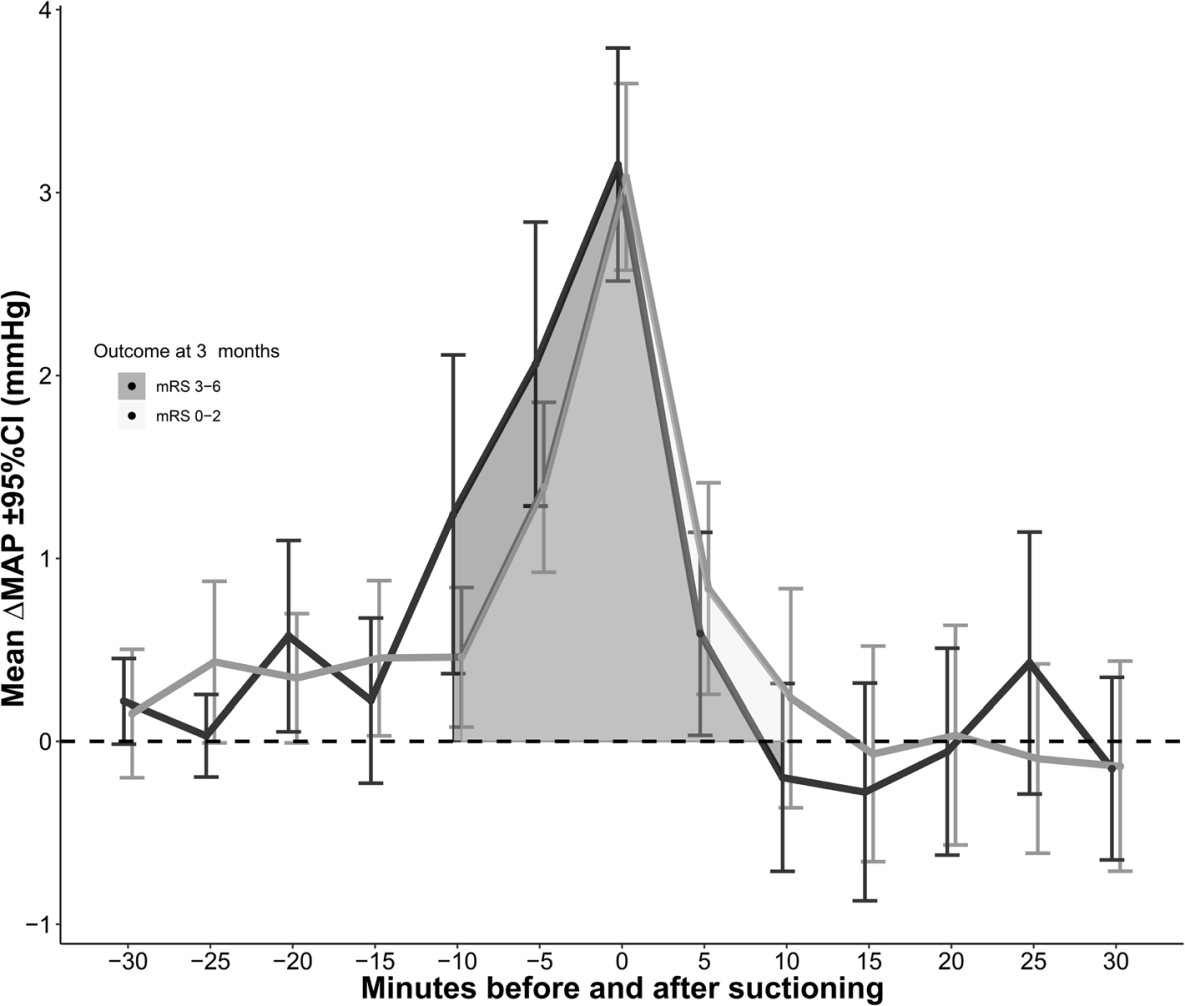
The increase of mean arterial pressure (MAP) during endotracheal suctioning (total *N* = 1080 episodes) was not significantly different across patients with good or poor outcome (*P* > 0.2). mRS, modified Rankin Scale Score

∆MAP was similar across patients’ outcomes (adjusted for midazolam, *p* = 0.263; or RASS, *p* = 0.344; Fig. 4).

## Discussion

The main findings of this study are that hemodynamic changes can be quantified during endotracheal suctioning and that a less pronounced increase in heart rate is associated with delayed arousal and worse functional outcome.

To the best of our knowledge, this is the first study systematically investigating the hemodynamic response to ES in SAH patients. ES provokes a cumulative hemodynamic response by activation of sympathetic, parasympathetic, and cough-related neurons [4, 20]. The autonomic response reflects a complex interaction between the brain and cardiovascular system. Besides the cardiovascular intrinsic system and the endocrine system, the autonomic nervous system (ANS) regulates heart rate and blood pressure. The ANS in turn is controlled by the sympathetic and parasympathetic system. Well-known brain-heart interactions during brain injury include acute myocardial injury, ECG changes, and arrhythmias which are commonly observed in SAH patients [21]. Moreover, mounting evidence suggests that a cerebral origin of cardiac dysfunctions results from activation of the hypothalamic–pituitary–adrenal (HPA) axis, catecholamine surge, and sympathetic and parasympathetic regulation [22].

Our analysis showed that poor-grade patients had less pronounced increases in HR during ES. This might indicate that a certain degree of connectivity is warranted for the hemodynamic response. First, the preservation of autonomic pathways involved in the transmission of the stimuli is needed. While cardiac sympathetic preganglionic nerves originate from the upper thoracic part of the spinal cord (T1–T4), parasympathetic neurons emerge from the mid-brain, pons, and medulla oblongata [23]. The intact interaction between descending inhibitory pathways and excitatory autonomic centers is important for control of cardiac function [24].

ES is associated with nociception [5] leading to sympathetic autonomic reactions including increases in heart rate, blood pressure, respiratory rate, sweating, vasoconstriction, and pupillary dilation [6, 7]. A recent study found that the nociceptive response to ES could be predicted using pupillary pain index measurements in patients with brain injury [5]. The nociceptive stimulus during ES is generally diminished by sedative and analgesic drugs. Still, this sympathetic stimulus may in addition provide insights into the integrity of associated brain regions. The central pain network involves several supraspinal areas including the thalamus, anterior cingulate cortex, amygdala, insula, primary and secondary somatosensory cortices, prefrontal cortices, and the periaqueductal gray as assessed by neuroimaging and electrophysiological studies [25, 26]. Interestingly, we could not find any association between HR increase and radiographic parameters as assessed by the modified Fisher score, SEBES, or intraparenchymal hemorrhage on admission.

Besides sympathetic activation, tracheal stimulation may result in an elevated parasympathetic drive as a consequence of activated vagal afferent nerves with subsequent bradycardia and hypotension [9].

Apart from autonomic reactions, mechanical stimulation of the airway mucosa provokes cough-related afferents via the vagal nerve which project to the brainstem respiratory network before a cough motor pattern is generated [4]. The cough reflex itself is again associated with an increase in HR [8]. Therefore, a higher HR increase might indicate the integrity of brainstem function. However, our data do not provide information whether a cough reflex was triggered during ES in our patients.

Notably, hypoxemia with the consequence of a decrease in HR secondary to a vagally mediated reflex [27] might have had a minor effect on HR in our patients based on the protocol for preoxygenation before ES.

Overall, the cumulative HR response was positive. In line with underlying pathophysiologic mechanisms, different responses of HR during ES could be identified. Interestingly, when separating in increasing and decreasing HR during ES, only positive but not negative HR responses discrimintated between good and poor functional outcome.

Most of our patients were sedated when ES was performed in the first 72 h after admission which is reflected by a RASS of − 4 or − 5 during ES. This may have attenuated the hemodynamic response observed. Therefore, different sedation levels might have influenced our results. Even after adjusting the multivariable model for the level of sedation (midazolam dose) and the level of consciousness (RASS), our results were suggestive of different patterns of ∆HR.

The observed cumulative increase in HR during ES was minor. Based on our approach to analyze 5 min-averaged data of hemodynamic monitoring, we might have underestimated the true effect of suctioning on HR and MAP. Still, there was a clear hemodynamic effect with discrimination of patients with prolonged decreased level of consciousness and worse functional outcome at 3 months.

Estimation of the time to arousal is challenging. Clinical evaluation and automated quantification of brain stem reflexes including pupillary light reflex and vestibulo-ocular reflex [28] could add to the prognostic value of comatose patients [29]. In a recent study conducted in patients after cardiac arrest, reduced quantitative pupillary light reflex performed well in mortality prediction [30]. Surface EEG in unresponsive patients may help to identify patients with brain activity despite missing motor behavior [3]. Besides that, neurophysiological studies with evoked potentials and brain imaging such as MRI are used to estimate patients’ outcomes [29]. Testing of ANS is a further cornerstone in prognostication of severely brain-injured patients. Several methods such as quantitative pupillometry [31] and heart rate variability [32] have been tested with promising results. Importantly, ES is a clinical routine procedure. Our results indicate that the extent of HR responses to ES in the very early phase after SAH may be useful to discriminate patients with delayed awakening. Time to arousal is determined by the amount of brain injury and pharmacological treatment. Notably, all patients included in our study were weaned off sedation in the ICU.

## Limitations

Several limitations deserve to be mentioned. First, the retrospective analysis of prospectively collected data does not prove causality. Our findings are hypothesis-generating and should be interpreted in the setting of the disease and the need for pharmacological treatment. It is important to mention that muscle relaxation medication was not applied in any patients during or before ES. Second, ES is a nociceptive stimulus routinely performed in mechanically ventilated patients. Consequently, good-grade patients either not requiring mechanical ventilation or those who were extubated immediately after the intervention (coiling/clipping) without ES procedure performed were not included in the current study. Third, time until regaining consciousness was evaluated by improvement of RASS. Time to coma recovery assessed by a coma recovery scale would have been interesting. Fourth, we did not account for the administration of anticholinergic or beta antagonist medications and irregular heart rhythms. However, these drugs from premedical history were discontinued in the early phase after SAH in patients requiring mechanical ventilation.

## Conclusion

Our results indicate that augmentation in HR may be considered to quantify the hemodynamic response during ES in brain-injured patients. Moreover, this biomarker may early discriminate patients with prolonged unconsciousness and worse outcome after SAH. Prospective studies are needed to understand the role of the autonomic system in prognostication using modern multimodal methods.

## Data Availability

The datasets used and/or analyzed during the current study are available from the corresponding author on reasonable request.

## Abbreviations

SAH: Subarachnoid hemorrhage
ES: Endotracheal suctioning
H&H: Hunt and Hess
mRS: Modified Rankin Scale
HR: Heart rate
MAP: Mean arterial blood pressure
CT: Computed tomography scans
mFisher: Modified Fisher score
SEBES: Subarachnoid Hemorrhage Early Brain Edema Score
DCI: Delayed cerebral ischemia
RASS: Richmond Agitation Sedation Scale
GEE: Generalized estimating equation models
ECG: Electrocardiogram
ANS: Autonomic nervous system
